# Detection of liver and spleen stiffness in rats with portal hypertension by two-dimensional shear wave elastography

**DOI:** 10.1186/s12880-022-00786-6

**Published:** 2022-04-13

**Authors:** YongJian Chen, JingYun Li, Qin Zhou, GuoRong Lyu, ShiLin Li

**Affiliations:** 1 Department of Ultrasound, The Second Affiliated Hospital of Fujian Medical University, No. 34 North Zhongshan Road, Licheng District, , Quanzhou, 362000 Fujian China; 2 Maternal and Child Health Service Application Technology Collaborative Innovation Center, Quanzhou Medical College, Quanzhou, Fujian China

**Keywords:** Portal hypertension, Two-dimensional shear wave elastography, Spleen stiffness, Liver stiffness, Hemodynamics

## Abstract

**Background:**

The measurement of liver stiffness (LS) and spleen stiffness (SS) based on ultrasound elastography can be used for non-invasive assessment of portal hypertension (PH). However, there are few studies on the corresponding mechanism of increased spleen stiffness. Our aim was to use two-dimensional shear wave elastrography (2D-SWE) to evaluate the relationship between LS and SS and the severity of PH in rats. And explore the mechanism of the increase of LS and SS in PH.

**Methods:**

Sixty male Sprague–Dawley rats were randomly divided into portal hypertension (PH group, n = 45) and normal control (NC group, n = 15). At 12 weeks, LS and SS was detected by 2D-SWE in vivo. Related hemodynamic parameters and portal vein pressure (PVP) was measured. Spleen and liver 2D-SWE detection was performed again after sacrifice. Pathological changes were observed.

**Results:**

The SS and LS were increased in PH group (*P* < 0.05). The SS decreased after sacrifice, and what's more the magnitude of SS decline significantly higher in PH group than in NC group (*P* < 0.05). The correlation between SS and PVP is stronger than LS (*r* = 0.624, *P* < 0.001). SS has positive correlation with indexes of hyperdynamic circulation, but LS was weakly. The correlation between SS and the pathological grade (*r* = 0.633, *P* < 0.001) was lower than that in LS (*r* = 0.905, *P* < 0.001). Multiple linear regression analysis revealed that SS, portal vein inner diameter (PVD) and splenic vein blood flow velocity (SVV) were significantly associated with PH.

**Conclusions:**

Spleen and liver measurement by 2D-SWE may be helpful in evaluating PVP. The correlation between SS and PVP is stronger than LS in rats measured by 2D-SWE. Hemodynamic circulation are important in the elevation of SS with portal hypertension. Pathological changes also have a degree of influence, but have more significance for the elevation of LS. SS may be a more effective noninvasive predictor of PH than LS.

## Background

Portal hypertension (PH) refers to the obstruction of blood flow and/or increased blood flow in the portal vein system under the action of various etiologies, resulting in a continuous increase in the pressure of the portal vein and its tributaries. It is a complex clinical syndrome. Ascites, splenomegaly, and collateral circulation formation and opening are the three common clinical manifestations of PH. And at the present, the most commonly used clinical evaluation is hepatic venous press gradient (HVPG) [1]. It is a complicated, expensive, and invasive test available only in specialized centers, which subjects have a poor tolerance [2]. Therefore, non-invasive assessment of the degree of PH is a critical research topic. Recent studies have shown that ultrasound elastography measurement of liver (LS) and spleen stiffness (SS) has good clinical application value in predicting esophageal varices in patients with cirrhosis and portal hypertension [3–6]. Among them, two-dimensional shear wave elastrography (2D-SWE) is an effective noninvasive diagnostic tool for predicting the presence of esophageal varices [4, 7], and is highly valuable in diagnosing clinically significant portal hypertension (CSPH) [8–10]. Some studies believe that SS has better evaluation of the severity of PH than LS[11], and other scholars believe that SS combined with LS is an excellent predictor of CSPH[12, 13]. Moreover, clinical studies and metanalysis have already shown that there is a close correlation between LS and HVPG when the value ≤ 10–12 mmHg, but above this threshold the strength of the correlation decreases markedly, and that SS reflects portal pressure better than LS [11, 14]. However, it is still unclear why the spleen elastic modulus increases in portal hypertension. To address this, this study used an animal model and two-dimensional shear wave elastrography (2D-SWE) to detect liver and spleen stiffness, and explored the relationship between LS, SS, and portal pressure, and hemodynamics and pathological changes, to evaluate the relationship between LS and SS in assessing the severity of portal hypertension.

## Methods

### Test object

Sixty Male Sprague–Dawley (145–195 g) rats were purchased from Shanghai Slack Laboratory Animal Co. Ltd. They were reared adaptively with standard basic feed for 7 days, and maintained at an appropriate temperature and humidity.

### Establishment of portal hypertension rat model

Using a completely random design, according to the experimental method of Königshofer [15], rats were divided into two groups, portal hypertension (PH group, n = 45) and normal control (NC group, n = 15). The PH group used the CCl_4_ (Sinopharm, Beijing, China) induction method to create the model. After local skin disinfection, a CCl_4_ corn oil solution (1 mL/kg body weight, twice a week) was injected subcutaneously, while the NC group was injected with corn oil only. During the experiment, the rats' general physical signs (coat color, mental state), movement flexibility, and body weight changes were observed and their body mass was measured weekly.

### SWE detection

**Fig. 1:**
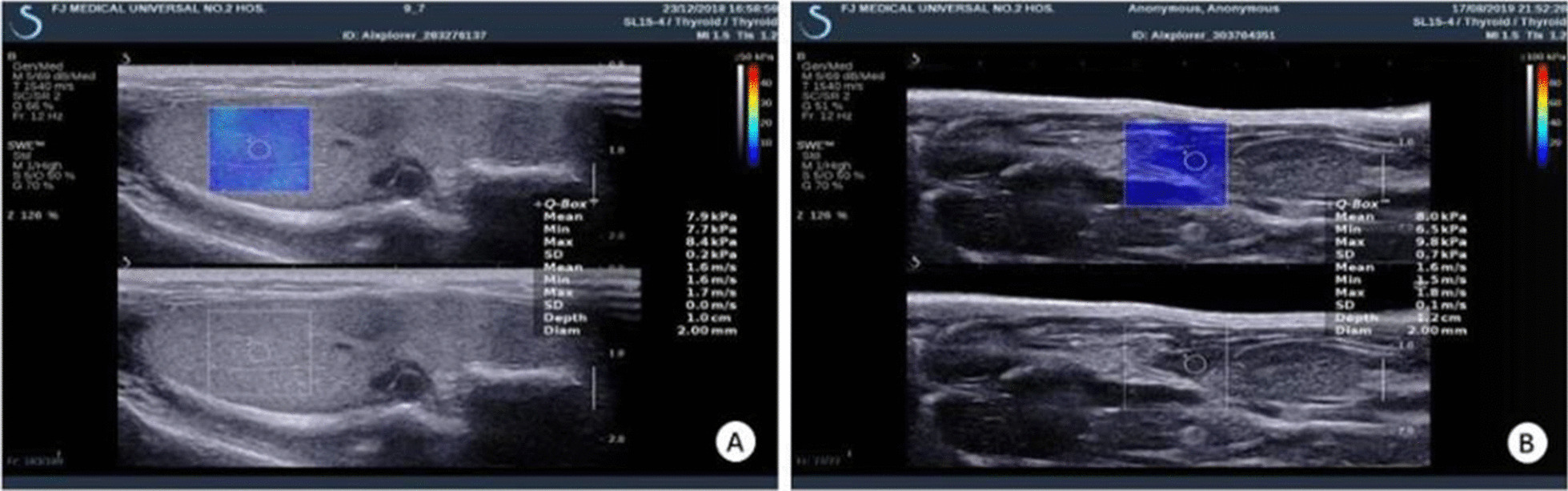
SWE measurement diagram. The sampling frame needs to be placed in the parenchyma while trying to avoid visible duct structures and keeping the probe fixed and vertical. **a** SWE measurement of the elastic modulus of the spleen. **b** SWE measurement of liver elastic modulus

As with previous experiments, rats were injected intraperitoneally with pentobarbital sodium (50 mg/kg) and after good anesthesia, the supine position and right supine position respectively were taken, and the diagnostic apparatus (Supersonic Imagine Aixplorer, Aix-en-provence, France) was used with a frequency 5–15 MHz linear array probe [16]. After the two-dimensional ultrasound, oblique section of the intercostal area clearly shows the liver or spleen, the sampling frame needs to be placed in the parenchyma while trying to avoid visible duct structures and keeping the probe fixed and vertical. The spleen (SS) or liver stiffness (LS) were measured 5 times at the same site, and the average was taken (Fig. 1).

### Detection of hemodynamic parameters

Color Doppler blood flow imaging was performed with a Mindray R70B ultrasonic diagnostic instrument using a 5–18 MHz high-frequency linear array probe. Measurements were made at the midpoint of the main portal vein, the splenic vein near the superior mesenteric artery, and the main portal vein and splenic vein in turn. The inner diameter (D) and peak velocity of blood (V) were observed, and the blood flow (Q) and congestion index (CI) were calculated using Q = 0.57πD^2^ V/4 × 60, and CI = πD^2^/(4 × 0.57 × V), respectively [17].

### Detection of portal pressure

The portal vein pressure (PVP) was measured by direct puncture through the main portal vein. The rat was taken in the supine position and after the limbs were fixed, the abdomen was depilated and disinfected. An incision was made about 3 cm along the midline of the abdomen to fully expose the hilar of the liver. A blunt glass minute needle was used to carefully dissociate the main portal vein and a 24 G indwelling needle was used to puncture the main portal vein. After the flow of blood begins, the other end of the indwelling needle is connected to the biological signal acquisition and analysis system through the blood pressure sensor. The PVP is recorded after the blood flow stabilizes.

### SWE analysis of LS and SS in sacrificed rats

The SWE measurements were repeated after injection of a large dose of anesthetic and confirmation of sacrificed of the mice.

### Pathological examination

The sampling operation was carried out after confirming the sacrificed of the mouse. The abdominal cavity was exposed and the morphology of the liver and spleen was observed. The organs were removed, fixed in formaldehyde, embedded in paraffin, serially sectioned, and HE staining, Masson staining, and electron microscopy were performed. According to the corresponding standards, the degree of liver fibrosis and splenic fibrosis are divided into 4 stages and 4 grades [18, 19].

### Statistical analysis

Measurement data was expressed as mean ± SD and SPSS (version 19.0; SPSS) was used for all analysis. Single-factor analysis of variance was used for comparison between groups, LSD test was used for pairwise comparison within groups, count data were expressed as cases or rates, and χ2 test was used for comparison between groups. A group t test was used to analyze the SS between live and sacrificed rats in the PH and NC groups, and the LS between live and sacrificed rats in the NC group. However, the subtraction of the value of LS between live and sacrificed rats in the PH group did not satisfy the normal distribution, and Wilcoxon signed rank test of paired samples was used. Spearman correlation analysis was used to analyze the correlation between the elastic modulus and the pathology of the liver and spleen, and a Pearson correlation analysis was used for the other correlation analyses. Multiple linear regression analysis was used to analyze factors affecting PH. *P* < 0.05 indicates a statistically significant difference.

## Results

### Portal pressure and pathological conditions

**Fig. 2:**
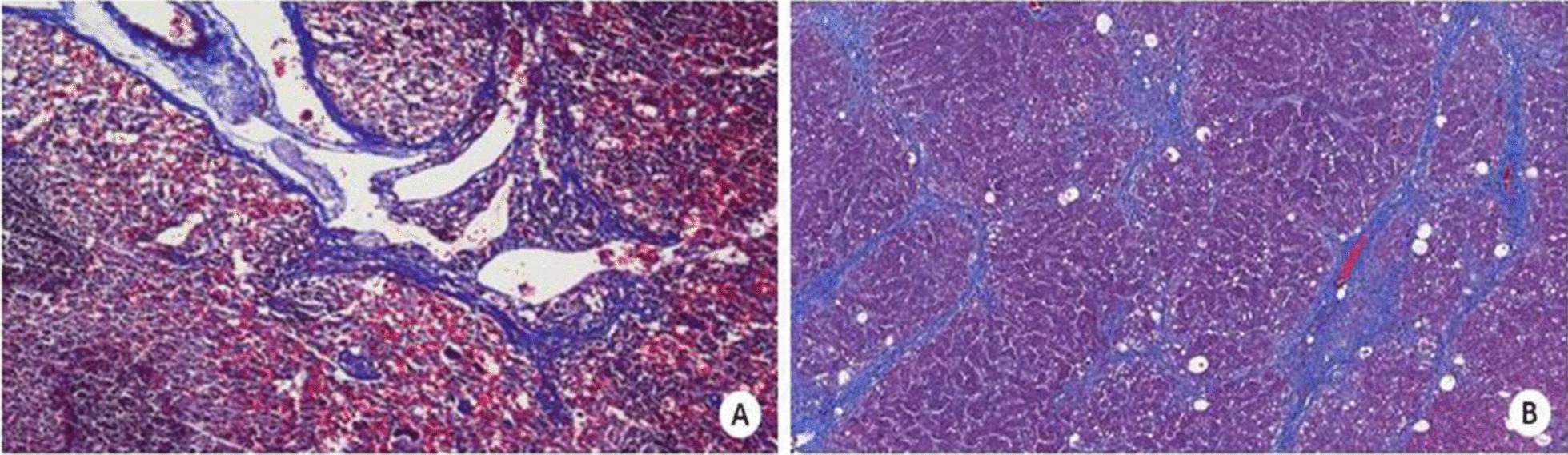
**a** Most splenic fibrosis is classified as Grade II (× 100). **b** Most stages of liver fibrosis are F4 (× 100)

The PVP was 11.38 ± 1.63 mmHg and 5.82 ± 0.65 mmHg in the PH and NC groups, respectively, and the difference between the groups was statistically significant (*P* < 0.05). The PH group showing a significantly higher PVP indicates that the model was successfully established. The pathological examination results of the liver and spleen are shown in Tables 1 and 2. The pathological grading of the spleen in the PH group was biased to grade II (Fig. 2A), and the pathological staging of the liver in the PH group was biased to stage F4 (Fig. 2B).

**Table 1:** Pathological classification of spleen

Group	0	I	II	III	IV	Total
Spleen in PH group	0	7	34	4	0	45
Spleen in NC group	13	2	0	0	0	15
Total	13	9	34	4	0	60

*PH* portal hypertension, *NC* normal control

**Table 2:** Pathological staging of liver

Group	S0	S1	S2	S3	S4	Total
Liver in PH group	0	2	3	12	28	45
Liver in NC group	15	2	3	0	0	15
Total	15	4	6	12	28	60

*PH* portal hypertension, *NC* normal control

### Analysis of elastic modulus of liver and spleen

The measurement results of the elastic modulus of the liver and spleen are shown in Table 3. (1) There was a statistically significant difference between the alive and post-sacrifice SS in the PH group (*t* = 11.513, *P* < 0.05). (2) There was a statistically significant difference between the alive and post-sacrifice LS in the PH group (Z = − 5.358, *P* < 0.05). (3) There was a statistically significant difference between the alive and post-sacrifice SS in the normal group (*t* = 3.829, *P* < 0.05). (4) There was no statistically significant difference between alive and post-sacrifice LS in the normal group (*t* = 3.238, *P* > 0.05).

**Table 3:** Rat liver and spleen elastic modulus (kPa)

Group	Living SS (X ± SD)	Post-sacrifice SS (X ± SD)	Living SS (Q1, Q3)	Post-sacrifice LS (X ± SD)
PH group	14.46 ± 2.95	10.92 ± 1.55	(7.95, 10.95)	9.25 ± 1.98
NC group	8.75 ± 1.73	8.21 ± 1.36	(7.70, 10.10)	5.24 ± 0.39

*SS* spleen stiffness, *LS* liver stiffness, *PH* portal hypertension, *NC* normal control

### Analysis of hemodynamic parameters

Table 4 shows the hemodynamic measurement results of portal vein and splenic vein.

**Table 4:** Hemodynamic parameters and vessel diameter (*X* ± *SD*)

Group	Portal vein (PV)				Splenic vein (SV)			
	D (cm)	V (cm/s)	Q(mL/min)	CI(cm ms)	D (cm)	V (cm/s)	Q (mL/min)	CI (cm ms)
PH group	0.18 ± 0. 02*	13.15 ± 2.28*	11.80 ± 3.02*	3.55 ± 0.75*	0. 12 ± 0. 02*	13.05 ± 2.09*	5.10 ± 1.45*	1.60 ± 0.56*
NC group	0.13 ± 0.01	12.23 ± 1.25	5.65 ± 0.87	1.96 ± 0.29	0.09 ± 0.01	10.17 ± 1.12	2.14 ± 0.36	1.08 ± 0.21

Comparison of hemodynamic parameters and vessel diameter in PH group with those in normal group *P < 0.05. D inner diameter, V peak velocity of blood, Q blood flow, CI: congestion index, PH portal hypertension, NC normal control

### Correlation analysis

SS, LS, and PVP were all positively correlated, and the correlation between SS and PVP (*r* = 0.746, *P* < 0.001) was higher than that between LS and PVP (*r* = 0.624, *P* < 0.001). (2) SS was positively correlated with splenic vein congestion index (*r* = 0.764, *P* < 0.001) and also positively correlated with splenic vein blood flow (*r* = 0.751, *P* < 0.001). (3) LS was positively correlated with portal vein congestion index (*r* = 0.724, *P* < 0.001), and weakly correlated with portal vein blood flow (*r* = 0.361, *P* < 0.05). (4) SS and LS were positively correlated with pathological grading, but the correlation between SS and pathological grading of spleen (*r* = 0.633, *P* < 0.001) was lower than that between LS and liver pathological staging (*r* = 0.905, *P* < 0.001).

### Multiple linear regression analysis

The original measurement of portal pressure (continuous variable) was used as the dependent variable, and 10 factors including SS, LS, portal vein inner diameter (PVD), splenic vein inner diameter (SVD), portal vein blood flow velocity (PVV), splenic vein blood flow velocity (SVV), portal vein blood flow (PVQ), splenic vein blood flow (SVQ), portal vein congestion index (PVCI), and splenic vein congestion index (SVCI) were used as independent variables. Multiple linear regression analysis (stepwise method) showed that SS, PVD, and SVV were significantly associated with PH (Table 5). The regression model was significant and could explain 69.2% of the total variation (*R*^2^ = 0.692, *F* = 42.023, *P* < 0.001).

**Table 5:** Multiple linear regression analysis of influencing factors of PH

Items	B	SE	β	*t*	*P*	B (95% CI)
Constant term	− 3.816	1.492	–	− 2.557	0.013	− 6.806 to − 0.826
SS	0.389	0.076	0.505	5.149	< 0.001	0.238–0.541
PVD	33.774	12.407	0.304	2.722	0.009	8.919–58.629
SVV	0.246	0.109	0.198	2.252	0.028	0.027–0.466

*SS* spleen stiffness, *PVD* portal vein inner diameter, *SVV* splenic vein blood flow velocity, *PH* portal hypertension

## Discussion

The use of SWE to detect portal hypertension has become a critical research area in recent years [20, 21]. However, due to clinical conditions, the elastic modulus of liver and spleen cannot be directly studied with the increase in portal pressure, and there are few studies on the corresponding mechanism of increased spleen stiffness. The previous studies showed that the LS of isolated pig liver detected by SWE increased as hepatic venous pressure increased [22]. Giunta found that spleen stiffness was significantly positively correlated with the portal and hepatic venous pressure gradients [23, 24]. Their studies have shown that the stiffness of related organs increases with the increased vascular pressure, and SWE can indirectly reflect the vascular pressure gradient. Consistent with previous studies [12, 20, 25], this study shows that both the SS and LS in rats with portal hypertension are elevated, and that they are positively correlated with the severity of portal vein pressure. What’s more, this study further demonstrate that 2D-SWE detection of SS can better reflect the severity of portal hypertension. We speculate that this may be related to the portal hyperdynamic blood flow state [26] and the inconsistency between the pathological changes of the spleen and liver caused by portal hypertension.

Blood flow and congestion index can reflect hemodynamic changes in patients with portal hypertension [27, 28] and are the main indicators of hyperdynamic blood flow theory. In this study, the portal vein blood flow (PVQ), splenic vein blood flow (SVQ), and congestion index of rats in the portal hypertension group were higher than those in the control group [29, 30]. The ratio of SVQ to PVQ in the portal hypertension group also increased, indicating that the simulation of the hyperdynamic circulatory state dominated by splenic circulation was successful [31, 32]. In this study, SS was positively correlated with splenic vein congestion index and SVQ. The hyperdynamic circulatory state disappeared after the rats were sacrificed, and the SS of the normal and portal hypertension groups decreased compared with the living body, but the SS of the portal hypertension group decreased significantly. The SS is greater in the PH group compared to the NC group, so we speculate that the high dynamic circulation state during portal hypertension may be an important factor in the increase in spleen stiffness. However, it is worth noting that portal vein blood flow velocity (PVV) was increased in our study, but the degree of increase was not large and other effective indicators were basically consistent with the previous models [33]. We speculate the possible reasons are as follows: Firstly, it may be due to the error of the measurement method during the experiment. Secondly, we observed that some model rats have ascites, abdominal cavity stickiness and other symptoms during the experiment, which may be due to the abdominal cavity injection molding. We speculated that these manifestations may have an effect on the elevation of PVV.

Some scholars found that HVPG decreased after intrahepatic portal shunt via internal jugular vein. At the same time, SS decreased significantly, but LS had no statistical significance [34]. In this study, after sacrifice, the hyperdynamic circulatory state disappeared, and the change of LS was not as obvious as that of SS. The correlation between LS and portal vein congestion index and PVQ was lower than that between SS and splenic vein congestion index and SVQ. There are several clinical studies which suggest that SS measurements can more accurately evaluate the treatment of portal hypertension, and esophageal varices [35–37]. This suggests that SS may be more sensitive to the hyperdynamic circulatory state caused by portal hypertension than LS which can be explained by Poiseuille's law [38]. As the portal venous system is further away from the liver, the blood flow resistance decreases and the hyperdynamic circulation becomes more pronounced. Compared to the portal vein, the splenic vein is further from the liver, the resistance is smaller, and the hyperdynamic circulation becomes more obvious [39].

Liver fibrosis is one of the initiating factors leading to the increase of portal system resistance and aggravating PH [40, 41]. In this study, LS and SS are positively correlated with fibrosis classification, indicating that fibrosis is one of the reasons for the increase of LS and SS [42–45]. However, most splenic fibrosis can only reach level II, while liver fibrosis can reach stage F4. The pathological changes in the liver and spleen are not consistent and the correlation between LS and pathological changes is greater than that of SS, indicating that fibrosis is more significant for the elevation of LS. In comparison, hyperdynamic circulation is more relevant when considering the increase in SS.

In summary, SS and LS are both closely related to hyperdynamic circulation and fibrosis. We speculate that the obvious increase of SS compared to LS during portal hypertension is closely related to the state of hyperdynamic circulation. The spleen is the "elastic heart" of the portal venous system, acting as a pressure regulating hub to protect the liver and portal venous system from excessive overflow. Therefore, the spleen is more sensitive to increased pressure in the portal system and hyperdynamic circulatory states [46]. The liver is also strong in terms of self-regulation and anatomical factors such as ligaments on the surface weaken the influence of hemodynamics. Therefore, the correlation between SS and PVP determined by SWE is higher than LS in this study. In addition, our study also found that SS was significantly associated with PH based on multiple linear regression analysis. Combined with previous studies, we speculate that SS may be a more suitable method to supplement or replace HVPG than LS, especially in the follow-up of high-risk patients with portal hypertension [47, 48].

## Conclusions

In conclusion, this study shows that, as determined by SWE, the correlation between SS and PVP is higher than that of LS and PVP. This study also shows that the hemodynamic changes of the highly dynamic circulation with the splenic circulation as an important component, leads to a higher correlation between portal pressure and SS than LS. Fibrosis may play a more important role in the increase of liver stiffness rather that an increase of spleen stiffness in regard to portal hypertension. Moreover, SS may be a more effective noninvasive predictor of PH than LS.

## Data Availability

The datasets generated and analyzed during the current study are not publicly due to privacy restrictions but available from the corresponding author upon reasonable request.

## Abbreviations

PH: Portal hypertension
HVPG: Hepatic venous press gradient
LS: Liver stiffness
SS: Spleen stiffness
2D-SWE: Two-dimensional shear wave elastrography
CSPH: Clinically significant portal hypertension
TE: Transient elastography
PVD: Portal vein inner diameter
SVD: Splenic vein inner diameter
PVV: Portal vein blood flow velocity
SVV: Splenic vein blood flow velocity
PVQ: Portal vein blood flow
SVQ: Splenic vein blood flow

## References

[1] Berzigotti A, Seijo S, Reverter E (2013). Assessing portal hypertension in liver diseases. Expert Rev Gastroenterol Hepatol.

[2] Karagiannakis DS, Voulgaris T, Siakavellas SI (2018). Evaluation of portal hypertension in the cirrhotic patient: hepatic vein pressure gradient and beyond. Scand J Gastroenterol.

[3] Fofiu R, Bende F, Popescu A (2021). Spleen and liver stiffness for predicting high-risk varices in patients with compensated liver cirrhosis. Ultrasound Med Biol.

[4] Kim TY, Kim TY, Kim Y (2016). Diagnostic performance of shear wave elastography for predicting esophageal varices in patients with compensated liver cirrhosis. J Ultrasound Med.

[5] Hu X, Huang X, Hou J (2021). Diagnostic accuracy of spleen stiffness to evaluate portal hypertension and esophageal varices in chronic liver disease: a systematic review and meta-analysis. Eur Radiol.

[6] Elkrief L, Rautou PE, Ronot M (2015). Prospective comparison of spleen and liver stiffness by using shear-wave and transient elastography for detection of portal hypertension in cirrhosis. Radiology.

[7] Grgurević I, Bokun T, Mustapić S (2015). Real-time two-dimensional shear wave ultrasound elastography of the liver is a reliable predictor of clinical outcomes and the presence of esophageal varices in patients with compensated liver cirrhosis. Croat Med J.

[8] Stefanescu H, Rusu C, Lupsor-Platon M (2020). Liver stiffness assessed by ultrasound shear wave elastography from general electric accurately predicts clinically significant portal hypertension in patients with advanced chronic liver disease. Ultraschall Med.

[9] Jeon SK, Lee JM, Joo I (2020). Two-dimensional shear wave elastography with propagation maps for the assessment of liver fibrosis and clinically significant portal hypertension in patients with chronic liver disease: a prospective study. Acad Radiol.

[10] Procopet B, Berzigotti A, Abraldes JG (2015). Real-time shear-wave elastography: applicability, reliability and accuracy for clinically significant portal hypertension. J Hepatol.

[11] Ma XW, Wang L, Wu H et al. Spleen stiffness is superior to liver stiffness for predicting esophageal varices in chronic liver disease: a meta-analysis. PLoS ONE. 2016; 11: e0165786. 10.1371/journal.pone.0165786 PMC5102398 27829057

[12] Jansen C, Bogs C, Verlinden W (2017). Shear-wave elastography of the liver and spleen identifies clinically significant portal hypertension: a prospective multicentre study. Liver Int.

[13] Elkrief L, Ronot M, Andrade F (2018). Non-invasive evaluation of portal hypertension using shear-wave elastography: analysis of two algorithms combining liver and spleen stiffness in 191 patients with cirrhosis. Aliment Pharmacol Ther.

[14] Vizzutti F, Arena U, Romanelli RG (2007). Liver stiffness measurement predicts severe portal hypertension in patients with HCV-related cirrhosis. Hepatology.

[15] Königshofer P, Brusilovskaya K, Schwabl P (2019). Animal models of portal hypertension. Biochim Biophys Acta Mol Basis Dis.

[16] Zhou Q, Guo HX, Li JY, et al. Evaluation of liver fibrosis in rats with non-alcoholic fatty liver disease by shear wave elastography. J Clin Ultrasound Med. 2020;251(03): 7–10.

[17] Kutlu R, Karaman I, Akbulut A (2002). Quantitative Doppler evaluation of the splenoportal venous system in various stages of cirrhosis: differences between right and left portal veins. J Clin Ultrasound.

[18] Kleiner DE, Brunt EM, Van Natta M (2005). Design and validation of a histological scoring system for nonalcoholic fatty liver disease. Hepatology.

[19] Wu HH, Chen JL (2005). Analysis of the effect of splenic fibrosis on immune function. Chin J Hepatobiliary Surg.

[20] Zhu YL, Ding H, Fu TT (2019). Portal hypertension in hepatitis B-related cirrhosis: diagnostic accuracy of liver and spleen stiffness by 2-D shear-wave elastography. Hepatol Res.

[21] Gibiino G, Garcovich M, Ainora ME (2019). Spleen ultrasound elastography: state of the art and future directions—a systematic review. Eur Rev Med Pharmacol Sci.

[22] Wang HW, Shi HN, Cheng J, et al. Real-time shear wave elastography (SWE) assessment of short- and long-term treatment outcome in Budd-Chiari syndrome: a pilot study. PLoS ONE. 2018;13(5): e0197550. 10.1371/journal.pone.0197550 PMC5976180 29847588

[23] Giunta M, La Mura V, Conti CB (2020). The role of spleen and liver elastography and color-doppler ultrasound in the assessment of transjugular intrahepatic portosystemic shunt function. Ultrasound Med Biol.

[24] Colecchia A, Montrone L, Scaioli E (2012). Measurement of spleen stiffness to evaluate portal hypertension and the presence of esophageal varices in patients with HCV-related cirrhosis. Gastroenterology.

[25] Sharma P, Kirnake V, Tyagi P (2013). Spleen stiffness in patients with cirrhosis in predicting esophageal varices. Am J Gastroenterol.

[26] Takuma Y, Nouso K, Morimoto Y (2016). Portal hypertension in patients with liver cirrhosis: diagnostic accuracy of spleen stiffness. Radiology.

[27] Kayacetin E, Efe D, Doğan C (2004). Portal and splenic hemodynamics in cirrhotic patients: relationship between esophageal variceal bleeding and the severity of hepatic failure. J Gastroenterol.

[28] Moriyasu F, Nishida O, Ban N (1986). "Congestion index" of the portal vein. AJR Am J Roentgenol.

[29] Iwakiri Y, Shah V, Rockey DC (2014). Vascular pathobiology in chronic liver disease and cirrhosis—current status and future directions. J Hepatol.

[30] Groszmann RJ, Abraldes JG (2005). Portal hypertension: from bedside to bench. J Clin Gastroenterol.

[31] Bosch J, Groszmann RJ, Shah VH (2015). Evolution in the understanding of the pathophysiological basis of portal hypertension: How changes in paradigm are leading to successful new treatments. J Hepatol.

[32] Baik SK, Jee MG, Jeong PH (2004). Relationship of hemodynamic indices and prognosis in patients with liver cirrhosis. Korean J Intern Med.

[33] Deng G (2013). Amelioration of carbon tetrachloride-induced cirrhosis and portal hypertension in rat using adenoviral gene transfer of Akt. World J Gastroenterol.

[34] Buechter M, Manka P, Theysohn JM (2018). Spleen stiffness is positively correlated with HVPG and decreases significantly after TIPS implantation. Dig Liver Dis.

[35] Marasco G, Dajti E, Ravaioli F (2020). Spleen stiffness measurement for assessing the response to β-blockers therapy for high-risk esophageal varices patients. Hepatol Int.

[36] Tseng Y, Li F, Wang J (2018). Spleen and liver stiffness for noninvasive assessment of portal hypertension in cirrhotic patients with large esophageal varices. J Clin Ultrasound.

[37] Attia D, Schoenemeier B, Rodt T (2015). Evaluation of liver and spleen stiffness with acoustic radiation force impulse quantification elastography for diagnosing clinically significant portal hypertension. Ultraschall Med.

[38] Li T, Yang Z (2005). Research progress of vasculopathy in portal hypertension. World J Gastroenterol.

[39] Yin Xiaoyu Lu, Mingde HJ (2000). Portal hemodynamic changes and esophageal veins in portal hypertension of liver cirrhosis. Chin J Ultrasound Imaging.

[40] Turco L, Garcia-Tsao G (2019). Portal hypertension: pathogenesis and diagnosis. Clin Liver Dis.

[41] McConnell M, Iwakiri Y (2018). Biology of portal hypertension. Hepatol Int.

[42] Bende F, Sporea I, Şirli R (2020). The performance of a 2-dimensional shear-wave elastography technique for predicting different stages of liver fibrosis using transient elastography as the control method. Ultrasound Q.

[43] Lee DH, Lee ES, Lee JY (2020). Two-dimensional-shear wave elastography with a propagation map: prospective evaluation of liver fibrosis using histopathology as the reference standard. Korean J Radiol.

[44] Ferraioli G, Barr RG (2020). Ultrasound liver elastography beyond liver fibrosis assessment. World J Gastroenterol.

[45] Iimuro Y, Yada A, Okada T (2020). Cytoglobin-expressing cells in the splenic cords contribute to splenic fibrosis in cirrhotic patients. Histol Histopathol.

[46] Weinzirl J, Garnitschnig L, Scheffers T (2021). Splenic rhythms and postprandial dynamics in physiology, portal hypertension, and functional hyposplenism: a review. Digestion.

[47] Colecchia A, Ravaioli F, Marasco G (2018). A combined model based on spleen stiffness measurement and Baveno VI criteria to rule out high-risk varices in advanced chronic liver disease. J Hepatol.

[48] Colecchia A, Colli A, Casazza G (2014). Spleen stiffness measurement can predict clinical complications in compensated HCV-related cirrhosis: a prospective study. J Hepatol.

